# Accelerating restrictive cardiomyopathy after liver transplantation in a patient with familial amyloidotic polyneuropathy: a case report

**DOI:** 10.1186/1752-1947-2-35

**Published:** 2008-02-01

**Authors:** Jason Robin, Sheridan Meyers, Maher Nahlawi, Jyothy Puthumana, Jon Lomasney, David Mehlman, Vera Rigolin, Charles Davidson

**Affiliations:** 1 Department of Medicine, Division of Cardiology, Northwestern University Feinberg School of Medicine, Chicago, Illinois, USA; 2 Department of Pathology, Northwestern University Feinberg School of Medicine, Chicago, Illinois, USA

## Abstract

**Introduction:**

Hereditary amyloidodis is a rare disease process with a propensity to cause polyneuropathies, autonomic dysfunction, and restrictive cardiomyopathy. It is transmitted in an autosomal dominant manner, with disease onset usually in the 20s-40s. The most common hereditary amyloidogenic protein, transthyretin, is synthesized in the liver and lies on Chromosome 18. Over 80 amyloidogenic transthyretin mutations have been described, the majority of which are neuropathic and hence the common name, Familial Amyloidotic Polyneuropathy. Until 1990, the disease was intractable with a 5–15 year survival after diagnosis. The prognosis changed after the implementation of orthotropic liver transplantation as a treatment strategy which halts the synthesis of amyloidogenic transthyretin. This has now has been performed over 1300 times in 67 centers.

**Case presentation:**

We describe the case of a man of Irish ancestry with Familial Amyloidotic Polyneuropathy and no clinical history of cardiac involvement. Shortly after orthotropic liver transplantation, he developed congestive heart failure. He was subsequently diagnosed with an accelerating post-transplant restrictive cardiomyopathy due to amyloid infiltration.

**Conclusion:**

A liver transplant induced cardiomyopathy in Familial Amyloidotic Polyneuropathy can be observed in patients without any history of cardiac symptoms. All patients with Familial Amyloidotic Polyneuropathy should be followed after transplantation to assess for a deterioration in cardiac function.

## Introduction

Familial Amyloid Polyneuropathy (FAP) is a disease process that has been described to affect the cardiovascular system. Clinical manifestations include hypotension, conduction disturbances, and most problematic, restrictive cardiomyopathies [1]. The majority of those with FAP have bothersome neuropathies, and are spared significant cardiovascular complications. Orthotropic liver transplantation (OLT) has been shown to stabilize and at times improve the neuropathic symptoms. Based on the pathogenesis of FAP, if OLT is performed prior to any of the clinical manifestations of cardiac amyloidosis, the likelihood of a patient succumbing to an amyloid cardiomyopathy should be significantly decreased.

## Case presentation

**Figure 1:**
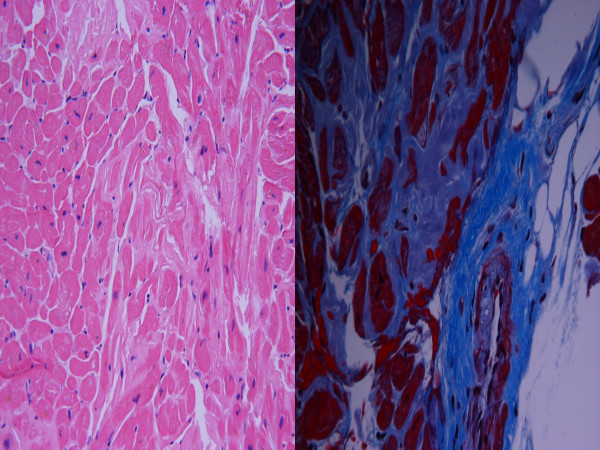
**a**. (Left) H&E. Higher power shot. Eosinophilic amorphous material separating myocytes with angulation of myocytes consistent with amyloidosis. **b**. (Right) Trichrome stain. Collagen around blood vessel (lower right corner) is blue, material surrounding myocytes is grey characteristic of amyloid. Interstitial material exhibited apple-green birefringence under polarized light with Congo red staining. (Not shown)

A 61 year old man presented with increasing dyspnea on exertion, ascites, and lower extremity edema. His medical history was remarkable for FAP which initially manifested ten years earlier as nausea and vomiting due to gastroparesis. In addition, he developed a painful peripheral neuropathy approximately two years later. Due to his progressive symptoms, he underwent OLT at another institution in August, 2006. Prior to his transplant, he had no cardiovascular complaints. His preoperative evaluation included a 2-dimensional echocardiogram. He did not have a cardiac catheterization. Approximately 2 months after his transplant, he began feeling dyspneic with mild to moderate activity. Shortly thereafter, he began to develop increasing abdominal girth and lower extremity edema. He presented to our institution in February, 2007 with further progression of these symptoms. His current medications included prednisone, tacrolimus, gabapentin, clotrimazole, valcyclovir, and pentamadine. His family history was significant for a father dying in his 50s due to gastrointestinal complications from FAP. On physical examination his heart rate was 100 with a blood pressure of 110/60 mmHg. He had an oxygen saturation of 97% while receiving oxygen at 4 L/min by nasal canula. He had no evidence of jaundice. He had crackles at the bases of his lungs bilaterally. His cardiovascular exam was remarkable for 10 cm of jugular venous distension, a regular rhythm with no murmurs, and an S4 gallop at the apex. His abdomen was mildly distended with shifting dullness to percussion and a liver edge 4 cm below the right costal margin. He had 2 + bilateral lower extremity edema. The ECG demonstrated sinus tachycardia with normal voltage, right axis deviation, and a left posterior divisional block. The Chest XRAY demonstrated mild cardiomegaly, patchy infiltration with associated atelectasis at the lung bases and a small right sided pleural effusion. The echocardiogram demonstrated an increase in left atrial volume, but was otherwise unchanged from his preoperative echocardiogram. (Table 1) This prompted an inferiorvenocavogram to evaluate for possible stenosis at the level of the anastomosis. The pressure gradient across the inferior vena cava anastomosis was unremarkable at 1–2 mmHg. There was no evidence of portal hypertension. However, the overall central venous pressure was elevated at 18–19 mmHg. It was decided at this time to perform a right and left heart catheterization. The coronary arteries were angiographically normal. The right and left heart pressures were elevated. (Table 2) His pressure tracings demonstrated no evidence of intrathoracic-intracardiac pressure dissociation or ventricular interdependence. Overall, this pattern was most consistent with restrictive physiology. An endomyocardial biopsy was performed which was consistent with cardiac amyloidosis. (Figure 1) Subsequently, it was learned that the patient's mutation was on the transthyretin (TTR) gene, Threonine60Alanine. He was started on an aggressive diuretic regimen and began to feel moderate relief. He is currently being closely followed as an out-patient and will be considered for cardiac transplantation if his symptoms cannot be controlled with medical management.

**Table 1:** Echocardiograms: Pre- and Post- Liver Transplant

*Variable*	*May, 2006*	*February, 2007*
***Ejection Fraction***	55%	50%
Interventricular Septal Thickness (cm)*	1.5	1.2
Posterior Wall Thickness (cm)*	1.2	1.4
***Left Ventricular Hypertrophy***	Moderate	Moderate
Left Atrial Volume Index (cc/m2)**	24	40
***Diastolic Dysfunction***	Grade 2	Grade 2
***Right Ventricular Systolic Pressure (mmHg)***	25	35
***>mild valvular regurgitation***	No	No
***Pericardial effusion***	No	Trivial to Small

*Normal Value 0.6–1.1 cm

**Normal Value <28 cc/m2

**Table 2:** Right Heart Catheterization Pressures

RA	RV	PA	PCWP	LV	AO
16	65/9	63/32	37	155/9	154/82

-RA = Right Atrium, RV = Right Ventricle, PA = Pulmonary Artery, PCWP = Pulmonary Wedge Pressure, LV = left Ventricle, AO = Aortic Pressure

-Pressures are recorded in mmHg

-Normal Mean Values: RA (3 mm Hg), RV (25/4 mm Hg), PA (25/9 mm Hg) PCWP (9 mm Hg), LV (130/8 mm Hg), AO (130/70 mm Hg)

## Discussion

Amyloidosis is a disorder resulting from the abnormal deposition of a particular protein in various tissues of the body. The four most common forms of amyloidosis are: **(1) ***light chain *due to immunoglobulins; **(2) ***secondary *which is seen in chronic inflammatory states such as rheumatoid arthritis; **(3) ***senile *which is typically seen in those over the age of 80; and **(4) ***heriditary*. There are at least eight different proteins that have been recognized to cause the hereditary amyloidoses [2]. Of these, the amyloidgenicTTR (ATTR) protein is the most common, with the Val30Met (Portuguese type) being the most prevalent mutation causing ATTR (80% of cases) [2]. The most common manifestation of ATTR is a neuropathy, but clinical manifestations vary depending on the location of the mutation. The treatment of hereditary amyloidosis is OLT, which limits further synthesis of the mutated protein, and thus halts further deposition in the organs. The Val30Met mutation typically presents with neuropathy, cardiac conduction disturbances, GI dysfunction, nephrosis and carpal tunnel syndrome. The eighty-plus nonVal30Met mutations in general have a greater propensity to cause cardiomyopathies [1]. In instances of a concomitant cardiomyopathy in the setting of FAP, simultaneous heart-liver transplantation has been performed with some success, though the numbers are small [3,4].

What we describe here is a man with no clinical manifestations of a cardiomyopathy prior to OLT who developed a progressive cardiomyopathy shortly after obtaining a normal liver. This clinical presentation of rapid and progressive cardiac amyloidosis following liver transplantation for FAP has been described by Stangou et al. His group evaluated 20 patients with FAP, 14 of whom underwent OLT, and compared their post-operative cardiac course with 10 traditional cirrhotic patients who underwent OLT. He found that in the group of FAP patients with OLT, there was a significant change in mean interventricular septal thickness in the 5 patients with nonVal30met mutations (15.2 mm progressed to 21.5 mm over 3 months, p < 0.05). There was no significant change in those with Val30Met mutations who underwent OLT, those with FAP who did not receive a liver or in the patients with traditional cirrhosis after OLT. In addition, 2 of the nonVal30Met patients were dead at 3 months due to heart failure [5]. Of interest, Olofsson et al also demonstrated progressive ventricular thickness in a group of FAP patients post-OLT with the more common Val30Met mutation, though the degree of ventricular hypertrophy was not as dramatic as was seen in Stangou's populatin (mean IVS thickness 11.5 mm before OLT and 13.1 mm at 18 months) [6].

The current hypothesis as to why this may occur has been looked at by previous investigators. Biochemical evidence suggests that before OLT, variant TTR-derived amyloid fibrils form a template to which wild type TTR attaches to after OLT. Yazaki et al studied 6 patients with FAP and evidence of cardiac involvement. Amyloid fibrils of the heart were composed of wild-type TTR as well as variant TTR at a ratio of about 1:1 in 5 patients without liver transplantation. In the patient with a transplanted liver, about 80% of the cardiac amyloid consisted of wild-type TTR [7]. He concluded that wild-type TTR contributes greatly to the development of amyloid deposition in the heart of FAP patients after OLT. This hypothesis is still considered by most experts to be the most likely explanation for the underlying pathophysiology. Interestingly, our patient unquestionably developed a rapid progressive cardiomyopathy following OLT, though with minimal change in overall myocardial thickness when compared to the pre-OLT echocardiogram. There was however a significant degree of change in left atrial volume. We postulate that the amyloid deposition may cause significant pathological changes on a cellular level leading to restriction, before there is macroscopic evidence of increasing ventricular thickness.

## Conclusion

Therefore, the results to date indicate that paradoxical wild-type TTR deposition after OLT can preferentially occur in myocardium, leading to even fatal cardiac dysfunction. What are the long-term implications? Essentially, the long-term outcome after OLT in FAP is still unknown. Although malnutrition, neuropathy, and renal insufficiency may be ameliorated, there is concern that an accelerating restrictive cardiomyopathy may limit survival. FAP patients with Val30Met and especially those with nonVal30Met mutations must be followed by echocardiography on a regular basis after OLT to enable an identification of individuals with increasing cardiomyopathy. However, as was seen in our patient, an accelerated cardiomyopathy may present clinically before there is echocardiographic evidence of amyloid deposition. This finding should urge physicians to measure cardiac hemodynamics in this setting, especially when there is no significant change in ventricular thicknesss. Echocardiography with Doppler can be useful, but in our case, the diagnosis was made with cardiac catheterization. With more data, combined heart and liver transplantation might be considered as an initial management strategy, even in those with a non existent or a *subclinical *cardiomyopathy. Alternatively, a new medical trial such as stabilization of wild-type TTR and/or reduction of the amount of substrate available for amyloid formation using antisense oligonucleotides may in the future be therapeutic options for FAP patients, before and after OLT [8-10].

## Abbreviations

1. FAP: Familial Amyloid Polyneuropathy

2. OLT: Orthotropic Liver Transplantation

3. TTR: Transthyretin

4. ATTR: AmyloidgenicTTR

## Competing interests

The author(s) declare that they have no competing interests.

## Authors' contributions

All authors were involved with the writing/reviewing of the manuscript. All authors approved the final manuscript.

## Consent

Written informed consent was obtained from the patient for publication of this case report and any accompanying images. A copy of the written consent is available for review by the Editor-in-Chief of this journal.
